# Influence of Serum Vitamin D on Age‐Related Physiological Changes: A Cross‐Sectional Study in Middle‐Aged Adults

**DOI:** 10.1002/cph4.70047

**Published:** 2025-09-02

**Authors:** Poulami Dhar, Prajna Bhandary, Shailaja Moodithaya

**Affiliations:** ^1^ Department of Physiology Nitte (Deemed to be University), KS Hegde Medical Academy Mangalore Karnataka India; ^2^ Central Research Laboratory Nitte (Deemed to be University), KS Hegde Medical Academy Mangalore Karnataka India

**Keywords:** aging, anthropometry, phenotypic marker, vitamin D, walking speed

## Abstract

**Background:**

The pace of physiological deterioration is variable among living beings. Vitamin D is proven to be one of the crucial yet deficient vitamins. Hypovitaminosis D is often marked by aging, but young adults are also not exempt. Thus, to identify the lacunae and bridge the gap between aging and vitamin D, this study selected the middle‐aged age group and their phenotypic aging markers. Physiological changes are gradual and measurable using non‐invasive methods, and they contribute to the phenotypic aging markers like BMI, WHR, *F*%, WS, and HGS. This study hypothesizes that the minute changes in phenotypic markers in young adults, which are prominent during middle age and potentially cause early aging, are influenced by Vitamin D.

**Methods:**

The study is based on a cross‐sectional design. Healthy individuals were recruited from the OPD following convenience sampling, and anthropometric and physical assessments were performed. Then, the blood samples were assessed for vitamin D levels. After segregating deficient (< 20 ng/dL), insufficient (20–30 ng/mL), and sufficient (> 20 ng/dL), the comparison was drawn.

**Result:**

A frequency analysis of three groups and comparisons among them was made. Association of phenotypic markers with vitamin D was shown.

**Conclusion:**

The present study population attempted to establish the hypothesis; however, chronological age can have multicollinearity with all variables, and sample size is a limitation of this study.

## Graphical abstract

The minute changes in physiological changes in young adults accumulate and become prominent during middle age, which potentially cause early aging, and may be influenced by serum Vitamin D. Hypovitaminosis D is dominating the world, so it is crucial to find whether chronological age or vitamin D is a determinant of biological aging.

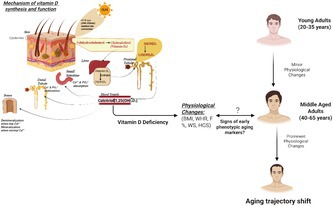

## 1 Introduction

Vitamins are essential micronutrients in the human body, and around seven different vitamins are present biologically. All these vitamins are required to successfully carry out physiological activities, including maintaining immunity, bone mineralization, skin health, normal vision, vascular health, etc. Vitamin D is one of these vitamins and possesses a pleiotropic role in biological actions (Gil et al. 2018; Verstuyf et al. 2010). Being a lipid‐soluble vitamin, it can mediate several physiological pathways by crossing the plasma membrane. Previous studies have documented vitamin D deficiency as one of the most common deficiencies around the globe, irrespective of the status of sun exposure. Vitamin D deficiency is often associated with the deterioration of bone health and causes diseases like rickets (in children), osteoporosis, and muscle loss. Apart from the conventional diseases, vitamin D deficiency is constantly being addressed in cardiovascular diseases, metabolic diseases, and neurological impairments. Since vitamin D can mediate its action through non‐genomic and genomic pathways, it acts as a secosteroid hormone in several biological cascades (Uberti et al. 2017; Haussler et al. 2011). It influences autophagy, mitochondrial activity, inflammation, cellular oxidative activity, DNA disorders, and epigenomes. Its biological actions are mediated by calcitriol or 1,25(OH)_2_D. However, the active form of vitamin D is not readily available in the system; it is synthesized from 7‐dehydrocholestrol, which is stored in the epidermal layer of the skin. A cascade of reactions is involved in the process of synthesis. In the first step, sunlight is required to accentuate the conversion of 7‐dehydrocholestrol to cholecalciferol (vitamin D2). This vitamin D2 migrates through the bloodstream and enters the liver for further conversion into 25(OH)D2, which further enters the proximal tubules of the kidneys via the bloodstream. In the presence of CYP27B1 enzymes in the proximal tubules, hydroxylation of vitamin D2 occurs to form 1,25(OH)_2_D_3_ or calcitriol. The product of this cascade, calcitriol, acts on the small intestine, distal tubules, and bones for Ca^2+^ & PO^2−^ absorption, reabsorption, and mineralization, respectively. This hormonally active 1,25(OH)_2_D mediates its action via VDR/RXR complex, which enters the nucleus to bind VDRE (Vitamin D Response Element) on the specific genomic site to activate transcription to translate target protein via mRNA (Gil et al. 2018; Haussler et al. 2011) Figure 1.

**FIGURE 1:**
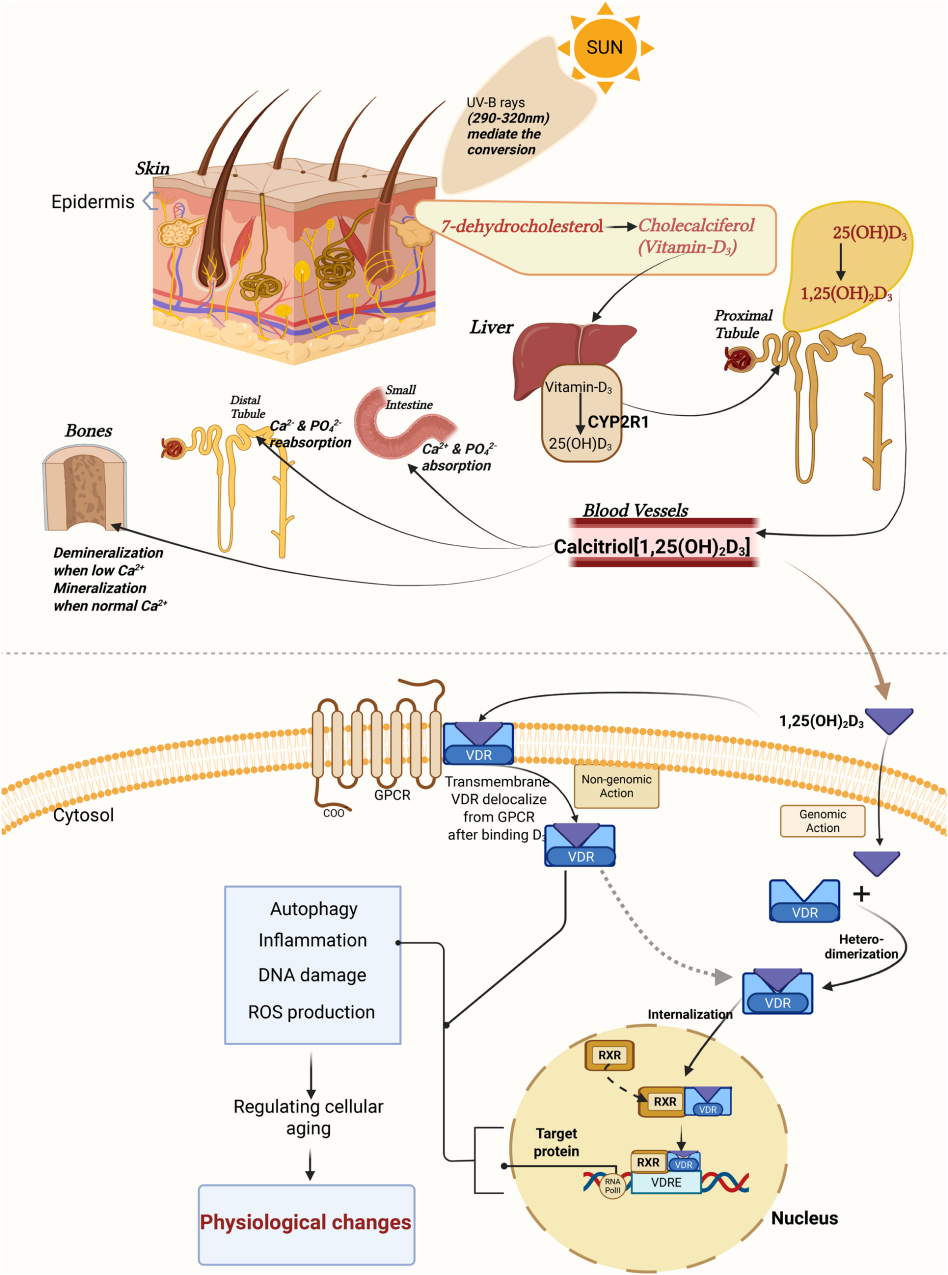
Mechanism of vitamin D synthesis from 7‐dehydrocholesterol in the presence of UVB sunrays. Conversion to calcitriol occurs through the liver and proximal tubules of the kidneys, and it has several functions in bones, the intestine, and the distal tubules of the kidneys. Its role in the cellular cascade is shown, which in turn influences physiological changes. Created in BioRender by Dhar Poulami (2025).

Berridge (2017) proposed a hypothesis that claims a connection between vitamin D and aging (Berridge 2017). This hypothetical conclusion was arrived at by Berridge due to the several biological actions that are orchestrated by calcitriol as a secosteroid hormone. Vitamin D regulates Ca^2+^, which in turn promotes autophagy via Ca^2+^ pumps and buffers. It activates TNF‐α to increase the expression of InsP3Rs, thereby regulating aging. In gerontological findings, mitochondrial dysfunction was addressed as a crucial factor for aging, while vitamin D functions to maintain the mitochondrial respiratory chain. In addition, there is a reduction in the expression of complex I of ETC, which affects the formation of ATP and generates ROS. These events cumulatively increase oxidative stress, and early aging can be considered. As a major regulator of oxidative quotient, vitamin D also mediates epigenetic landscapes via its multi‐genomic promoters by methylation, acetylation, sumoylation, etc. (Berridge 2017, 2016). Therefore, it can potentially regulate autophagy, inflammation, DNA damage, and ROS production, and hence cellular aging (Dhar et al. 2024). Progression in cellular aging is caused by modulating these cascades, resulting in physiological changes. Previous findings have raised the question of whether these changes are noticeable as a phenotypic marker (Hill et al. 2018; Meehan and Penckofer 2014; Elizondo‐Montemayor et al. 2017; Pourghaed et al. 2024). Phenotypic aging markers are permanent evidence of aging, often irreversible. The current study aims to bridge the gap between physiological changes and the occurrence of aging by identifying phenotypic aging markers and correlating them with vitamin D levels.

## 2 Methodology

### 2.1 Study Population and Sample

This study includes participants without chronic diseases to justify the occurrence of age‐related phenotypic changes in the middle‐aged population in the absence of comorbidities. Demographic data were collected in the OPD, and consented participants were taken to the examination room for anthropometric assessments, walking speed tests, and hand‐grip tests. Approximately 2 mL of a non‐fasting peripheral blood sample was collected in a no‐additive vacutainer for the segregation of serum. The serum sample was stored at −80°C for further experiments. The sample size was calculated based on 80% power, 5% alpha error, and an effect size of 0.5. Using G* power 3.1, the indicated sample size was 128. Due to the available data at the time of analysis, only 80 participants were included in the present study.

### 2.2 Study Design and Setting

This study employs a cross‐sectional design, comparing three groups of middle‐aged adults (40–65 years). Samples were collected from the General Medicine OPD based on conventional sampling and following middle‐aged healthy adults.

### 2.3 Data Procurement

#### 2.3.1 Anthropometric Measurements

Anthropometric measurements are the primary standards for non‐invasive study. They can be acquired by measuring physical parameters, including height, weight, waist‐hip circumference, fat percentage at triceps, biceps, subscapular, and suprailiac (Norton 2018). Additionally, walking speed and hand grip strength of any individual can be a window for assessment of functional health (McGrath et al. 2018; Amatachaya et al. 2020). These are the indices that were collected as phenotypic markers:

- Body mass index (BMI): Calculation of BMI will be carried out by the calculation $\mathrm{BMI}=\frac{W}{H^{2}}$, *W* = Weight (kg), *H* = Height (m). Here, a stadiometer will be used as the standard method to measure height to the nearest 0.01 m, and an electronic weighing machine will be used for weight measurement to 0.1 kg (Norton 2018).
- Waist‐to‐hip ratio (WHR): Measurement of waist circumference (WC) and hip circumference (HC) will be performed using a measuring tape in centimeters. WC measurement will be midway between the lower margin of the last palpable rib and the top iliac crest at the end of expiration, and HC will be around the waist area of the hip, with the feet held together (Norton 2018).
- Body fat percentage (*F%*): Harpenden caliper will be used for fat mass as well as fat‐free mass, considering the thickness of the skin folds, including the biceps, triceps, sub‐scapular, and supra‐iliac region (Norton 2018).
- Walking speed (WS): It was measured by using the 10‐m walk test under three trials, and the average was taken. A marked pathway of length 10 m was kept in the OPD, and consented participants were allowed to walk at their own pace. The initial 2 and the end 2 m were exempted for acceleration and deceleration. The time was considered in seconds (Amatachaya et al. 2020).
- Hand grip strength (HGS): Hand grip strength is a symbol of muscle strength, and reduced muscle strength represents aging. HGS was measured using Jamar's Hand Dynamometer, which is expressed in kg (McGrath et al. 2018).

#### 2.3.2 Blood Sample Analysis

Vitamin D was analyzed following the competitive ELISA method using the Reed Biotech Ltd. (RE10111) ELISA kit (Amatachaya et al. 2020). Serum 25‐hydroxyvitamin D was measured as the vitamin D level (Pourghaed et al. 2024):

1. VDD (vitamin D deficiency): vitamin D level < 20 ng/mL.
2. VDI (vitamin D insufficient): vitamin D level 20–30 ng/mL.
3. VDS (vitamin D sufficient): vitamin D level ≥ 30 ng/mL.

### 2.4 Statistical Analysis

SPSS 29.0 software (IBM Corp., Armonk, NY, USA) and Python 3.10 were used for data analysis and plotting (Fermín‐Martínez et al. 2023; Padilla et al. 2021). Normality of the variables was checked by the Shapiro–Wilk test, and data distribution was performed by frequency testing. Measurements of data were displayed in terms of mean ± standard deviation among the three groups. Comparison among the three groups was performed by using one‐way ANOVA. Pearson's correlation and Spearman's correlation were done depending on the data (parametric and non‐parametric). Multivariate linear regression was done to check the confounder and represented in the boxplot.

## 3 Results

### 3.1 Assessment of Vitamin D and Classified Into Three Groups

**FIGURE 2:**
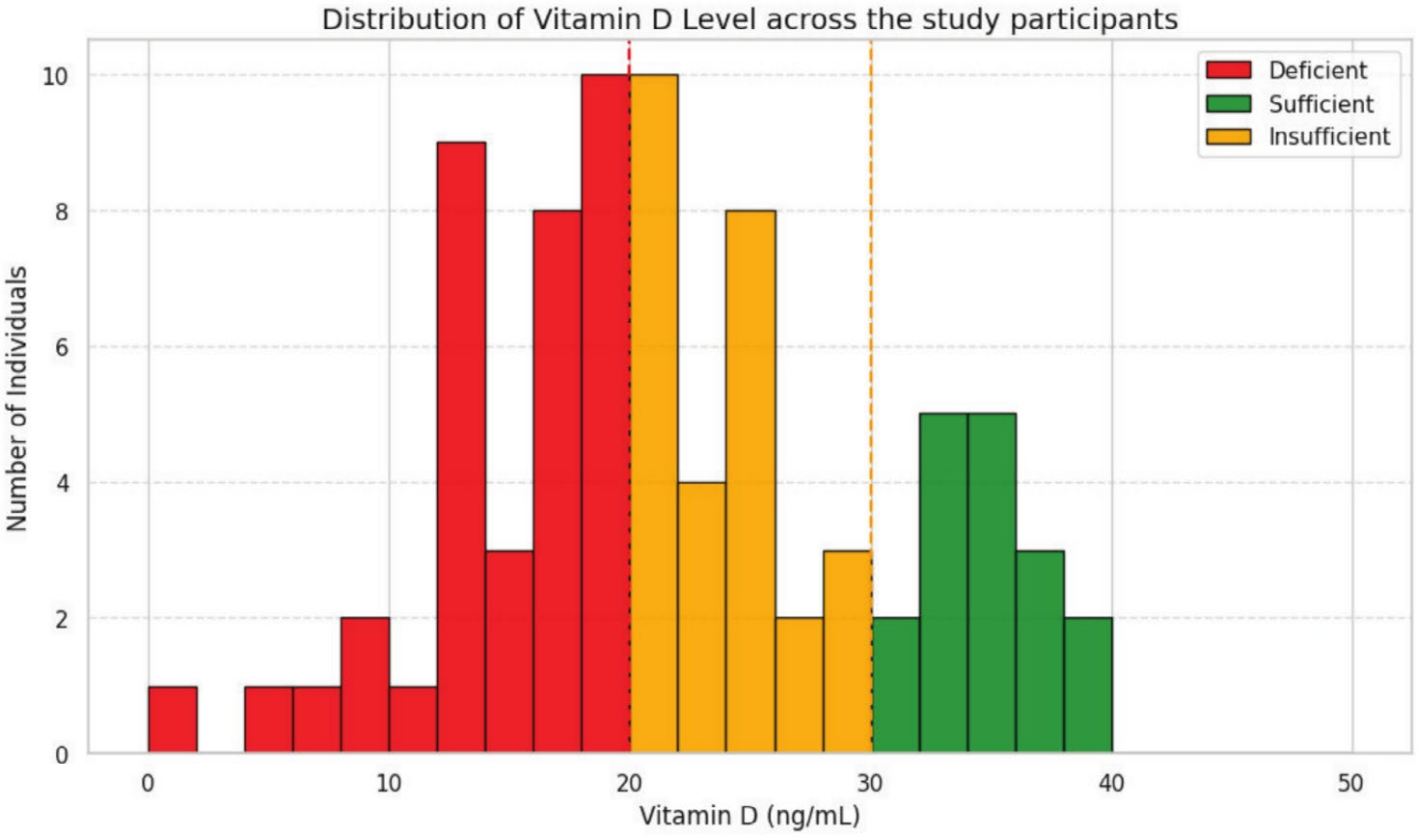
Frequency distribution of vitamin D level across the participants as deficient, insufficient, and sufficient. The *X*‐axis shows the serum vitamin D levels (ng/mL), and the *Y*‐axis shows the number of individuals (count).

On average, the mean VD level was 21.9 ± 8.5 ng/mL (*n* = 80; mean *±* SD). Vitamin D level was not normally distributed (Shapiro–Wilk test *p* > 0.05). Samples were stratified across all vitamin D categories, ranging from 1.49 to 39.3 ng/mL. Vitamin D values are plotted in a histogram, which shows asymmetrical distribution with right‐skewed and platykurtic (skewness: 0.16, kurtosis: −0.49). Stratification across vitamin D categories was group 1 (36%), group 2 (27%), and group 3 (17%). Overall, 63/80 (78.75%) fell under deficient and insufficient categories (Figure 2). No record for vitamin D toxicity > 150 ng/mL.

### 3.2 Association Between Phenotypic Aging Markers and Vitamin D

**FIGURE 3:**
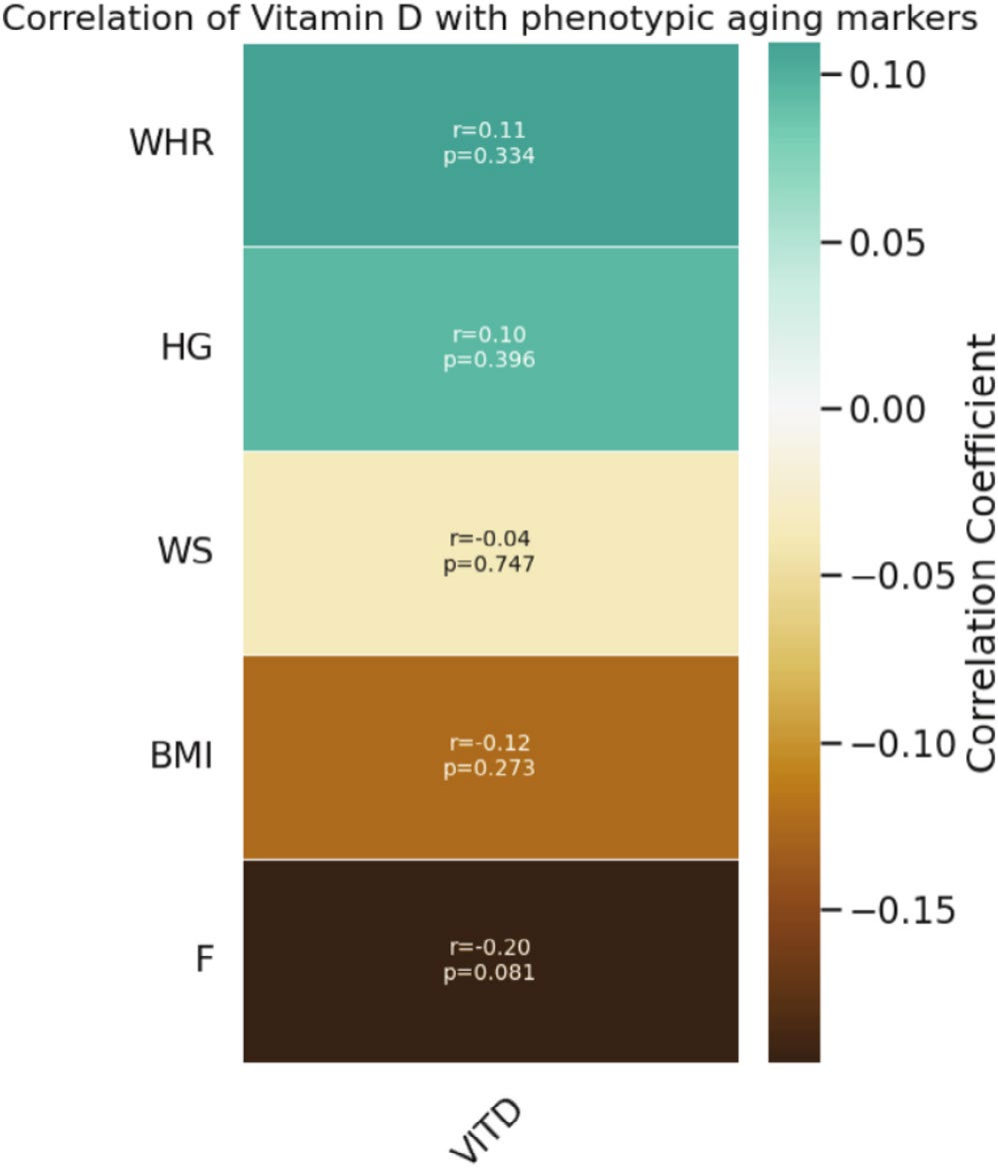
Heatmap showing correlation between vitamin D level and phenotypic aging markers. Pearson and Spearman correlations were drawn depending on the distribution of the variable.

Association of phenotypic markers was first observed with age distribution (age group 40–65 years). A trait of negative correlation with *F* (*r* = −0.07; *p* = 0.509), WS (*r* = −0.31; *p* = 0.005), HGS (*r* = −0.55; *p* = 0.0), and vitamin D (*r* = −0.14; *p* = 0.209) was obtained. On further target analysis between vitamin D and phenotypic aging markers, Pearson correlation for the parametric set and Spearman correlation for the non‐parametric set of markers were done. Phenotypic markers such as BMI (Spearman *r* = −0.12; *p* = 0.27), *F* (Pearson *r* = −0.20; *p* = 0.08), and WS (Spearman *r* = −0.04; *p* = 0.74) were negatively correlated (Figure 3). However, the correlation could not stand out as statistically significant at *p* < 0.05.

### 3.3 Comparison of Phenotypic Aging Markers Among the Three Groups

The phenotypic aging markers across the three groups are tabulated in mean ± SD in Table 1 below. Not all the phenotypic markers are normally distributed, but HGS (Shapiro–Wilk *p* = 0.04) and WHR (Shapiro–Wilk *p* = 0.00) are normally distributed, while BMI, *F*%, and WS are not normally distributed. Comparison of parametric tests using one‐way ANOVA shows no significance as *p* > 0.05. Similarly, non‐parametric tests using Kruskal–Wallis do not show significant (*p* > 0.05) differences in mean among the three groups.

**TABLE 1:** Comparison of mean phenotypic aging markers among the three study groups.

Parameters	VDD mean ± SD	VDI mean ± SD	VDS mean ± SD	*p*
Age (years)	53.16 ± 7.20	51.7 ± 6.41	51.64 ± 7.63	0.64
BMI (kg/m^2^)	23.86 ± 4.0	23.08 ± 3.84	22.82 ± 3.78	0.66
WHR (ratio)^a^	0.89 ± 0.06	0.90 ± 0.06	0.91 ± 0.05	0.61
WS (m/s)	0.75 ± 0.16	0.76 ± 0.12	0.74 ± 0.15	0.28
HGS (kg)^a^	52.36 ± 27.99	55.92 ± 28.25	59.41 ± 21.8	0.92
*F* (%)	9.58 ± 8.03	7.40 ± 8.07	6.47 ± 4.6	0.38

^a^ The means of parametric variables were compared by one‐way ANOVA.

### 3.4 Assessment of Confounders in Age‐Matched Distribution

**FIGURE 4:**
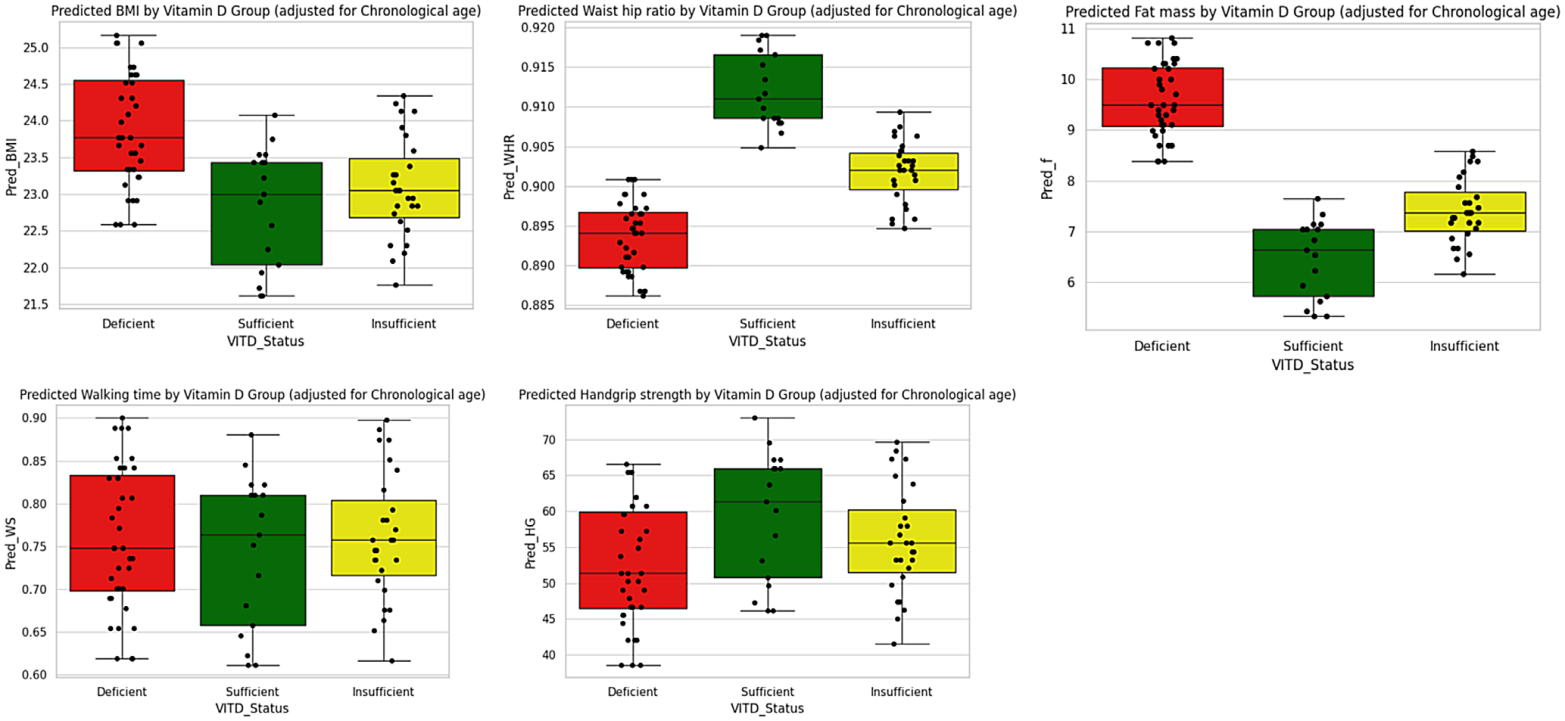
A diagrammatic representation of boxplots among the three study groups, adjusting for chronological age. After adjusting for chronological age, the model shows an improved difference among the means of the three study groups and phenotypic aging markers.

Ordinary least squares (OLS) method in multiple linear regression analysis uses the equation (*Y* = *β*_0_ + *β*_1_ *X*_1_ + *β*_2_ *X*_2_ + ⋯ + *β*_p_ *X*_p_ + *ϵ*) where *Y* represents the outcome, *X* represents the independent variable, and *ϵ* is the residual. Chronological age as a confounder was controlled, and multiple linear regression analysis was performed. Boxplot and strip plot visualize the effect and change in mean among the three groups (Figure 4).

## 4 Discussion

Frequency analysis was performed on collected samples to assess the vitamin D levels in the study samples and segregate them into three groups according to the cutoff. VDD, VDI, and VDS are three groups of the total collected samples. The frequency distribution shows 78.75% of middle‐aged adults fall into the deficient category, though chronic disease was an exclusion criterion. Chronic disease includes CKD, RF, DM, cirrhosis of the liver, and any neurological diseases. However, an analysis of the association between five phenotypic markers and vitamin D indicated that *F*, WS, and HGS were negatively correlated. In the case of WS and HGS, which usually reduce with age, they are also found to be significantly reduced irrespective of group comparison. A comparison study was performed among the three groups, VDD, VDI, and VDS, and phenotypic markers. Results do not show significance due to the asymmetric distribution of the sample. After all, the weight in the VDD group is the highest, and thus comparison was non‐significant. From another perspective, this indicates a further study to collect an even number of samples among different age groups, so significant insights can be drawn. Pragmatically, we accept chronological age as a true value of age, but it often deviates from biological age. Though the study group was age‐matched and exclusively free from chronic diseases, the study conducted an assessment of confounders using multiple linear regression analysis.

Present study provides a thorough investigation of chronological age, phenotypic aging markers, and serum vitamin D levels. The study participants were included in anthropometric assessments, physical assessments, and blood sample assays. Anthropometric facets are a replication of phenotypic aging markers like BMI, WHR, and *F*. Meanwhile, physical assessments like WS and HGS are also included in phenotypic aging markers. All these phenotypic markers are well studied in the research of gerontology. Most of the studies confirm the increase in BMI, WHR, and *F* while a decrease in WS and HGS with aging. Parallelly, studies on vitamin D claim deficiency causes increased BMI, WHR, and *F* while a decrease in WS and HGS (Fermín‐Martínez et al. 2023; Padilla et al. 2021). The study hypothesized that vitamin D deficiency may lead to the early onset of phenotypic changes concerning aging.

Here, we investigated the vitamin D level among middle‐aged adults. Though we selected the study in healthy participants to avoid the fact of comorbidities, we still achieved only 17% with sufficient vitamin D. That indicates the deficiency of vitamin D prevails even though there is no significant disease. Furthermore, we associated the level of vitamin D with phenotypic aging markers. BMI, WS, and *F* were negatively correlated with vitamin D, but the association is not statistically significant. On the other hand, WHR and HGS are not showing a correlation. Later, we examined each group of VDD, VDI, and VDS with phenotypic markers. This comparative study did not show a significant difference in mean among the three groups, which may be due to the uneven frequency.

**FIGURE 5:**
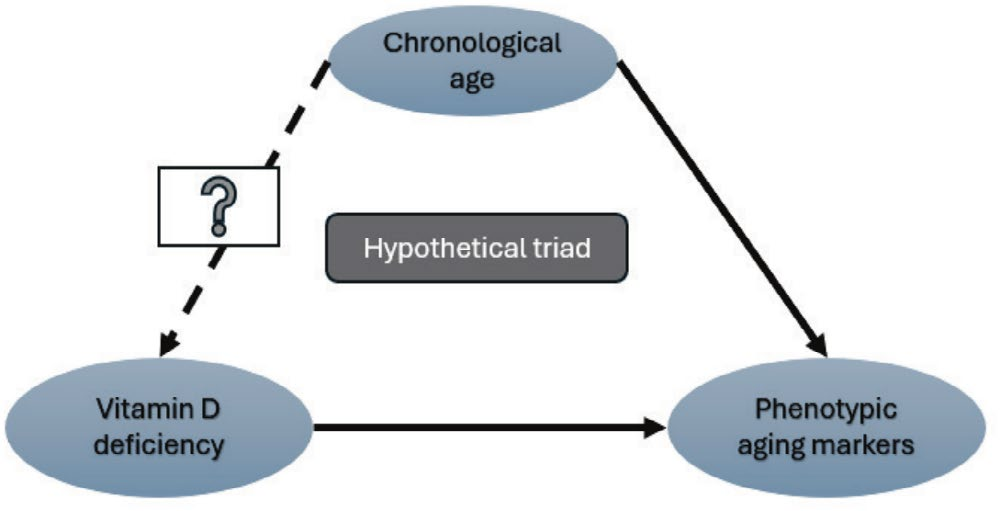
A hypothetical triad connecting the study relation. The triad expresses the relationship between vitamin D and the change in phenotypic aging markers, which is understood. At the same time, the association of the change in phenotypic aging markers and chronological aging is also significant. However, whether chronological aging will cause vitamin D deficiency among healthy individuals is unproven.

Additionally, multivariate analysis investigates chronological age as a confounder and makes the most of the relation. Often, a confounder is a variable that influences relationships with other variables. This analysis supports the fact that phenotypic aging can be seen with a chronological increase in age, and vitamin D deficiency can influence phenotypic aging. However, the role of chronological age in causing vitamin D deficiency is yet to be clear from this study (Figure 5). This study reflects a hypothetical triad, which explains that vitamin D deficiency may alter phenotypic aging markers, and with progression, age phenotypic markers change, but there is no evidence that vitamin D deficiency is assured with increased chronological age.

The critical viewpoint on this study is that the sample size is a limitation for establishing the hypothesis. In most the circumstances, middle‐aged adults were having one of the other comorbidities, and hence the sample size was a limitation. Secondly, the uneven distribution of the vitamin D sufficient population with gender bias can be another cause. However, this is a part of an ongoing project, and the hypothesis may be established after the completion of all the objectives of the proposal.

## 5 Conclusion

This study attempts to conclude that vitamin D influences physiological changes throughout life and visible phenotypic differences among different vitamin D groups. However, in most analyses, the associations did not meet statistical significance, possibly due to the limited sample size or homogeneity of study populations. A triad has been established in the discussion to show the possibility that chronological age is a confounder, which theoretically says that vitamin D deficiency might cause physiological changes, and an increase in chronological age might show physiological changes, but a clear statement regarding an increase in chronological age and a reduction in vitamin D cannot be made. The study population is free of chronic diseases but still has the most deficient individuals. This kind of study is advantageous in updating the aging research, where non‐significant results obviate the conventional practice. Future studies on epigenetic markers can evoke a significant status of cellular aging along with physiological changes.

## Author Contributions

**Poulami Dhar:** writing and analysis. **Prajna Bhandary:** laboratory assistance. **Shailaja Moodithaya:** drafting and review.

## Ethics Statement

Ethical approval was obtained by the Central Ethics Committee of Nitte Deemed to be University.

## Consent

A consent form was signed by the willing participants.

## Conflicts of Interest

The authors declare no conflicts of interest.

## Data Availability

Data will be available to the authors till the completion of the project.
